# Managing expectations, rights, and duties in large-scale genomics initiatives: a European comparison

**DOI:** 10.1038/s41431-022-01247-y

**Published:** 2022-12-06

**Authors:** Ruth Horn, Jennifer Merchant, The UK-FR GENE Consortium, Marion Abecassis, Mark Bale, Hervé Chneiweiss, Nina Hallowell, Angeliki Kerasidou, Anneke Lucassen, Jonathan Montgomery, Michael Parker, Christine Patch, Eva Winkler

**Affiliations:** 1 The Ethox Centre and Wellcome Centre for Ethics and Humanities, Nuffield Department of Population Health, University of Oxford, Oxford, UK; 2 Ethics of Medicine, Faculty of Medicine, University of Augsburg, Augsburg, Germany; 3 CNRS Law & Humanities/CERSA UMR-7109, University Paris-Panthéon-Assas, Paris, France; 4 Institut Universitaire de France, Paris, France; 5 DLA Piper Global Law, Paris, France; 6 Genomics England, London, UK; 7 CNRS, Inserm, Sorbonne University, Paris, France; 8 University of Oxford, Oxford, UK; 9 University College of London, London, UK; 10 Engagement and Society, Wellcome Connecting Science, Hinxton, UK; 11 University of Heidelberg, Heidelberg, Germany

**Keywords:** Medical ethics, Health policy, Genetics research

## Abstract

This article reports on the findings of an international workshop organised by the UK-France Genomics and Ethics Network (UK-FR GENE) in 2021. They focus specifically on how collection, storage and sharing of genomic data may pose challenges to established principles and values such as trust, confidentiality, and privacy in countries that have implemented, or are about to implement, large-scale national genomic initiatives. These challenges impact the relationships between patients/citizens and medicine/science, and on each party’s rights and duties towards each other. Our geographic scope of comparative analysis includes initiatives underway in England (Genomics England), France (Plan France Médecine Génomique) and Germany (German Human Genome-Phenome Archive). We discuss existing as well as future challenges raised by large-scale health data collection and management in each country. We conclude that the prospects of improving individualised patient healthcare as well as contributing to the scientific and research prosperity of any given nation engaged in health data collection, storage and processing are undeniable. However, we also attempt to demonstrate that biomedical data requires careful management, and transparent and accountable governance structures that are clearly communicated to patients/participants and citizens. Furthermore, when third parties partake as stakeholders, transparent consent protocols relative to data access and use come centre stage, and patient benefits must clearly outweigh commercial interests. Finally, any cross-border data transfer needs to be carefully managed to address incoherencies between regional, national, and supranational regulations and recommendations.

## Introduction

The implementation of data-driven healthcare in genomic medicine that decidedly relies on large health datasets challenges established boundaries between clinical interventions, research and other long-standing principles of healthcare such as trust, confidentiality, consent and privacy [1, 2]. Furthermore, large-scale genomic programmes have increasingly connected medicine’s primary goal of promoting health and preventing disease, to driving industry and economic growth.

These developments have led to calls to revisit the social contract between society and medicine/medical sciences [3–5], i.e., the explicit and implicit agreements between social groups or citizens and the government or any other governing actors or institutions [6]. The social contract that has been the subject of modern political theory (e.g., Thomas Hobbes, John Locke and Jean-Jacques Rousseau) is what establishes and delineates each party’s rights and duties towards each other and their reasonable mutual expectations. Consequently, it provides the basis of social order and trust between the individual and public institutions [7]. Changes to established norms and values challenge the social contract and the trust that originates from it, and hence may require that the contract be redefined.

An international workshop organised by the UK-France Genomics and Ethics Network (UK-FR GENE) in 2021, focused specifically on how collection, storage and sharing of genomic data may now present challenges to the social contract in countries that have implemented, or are about to implement, national genomic initiatives. In this paper, we report on the workshop discussions that centred on some of these challenges and the ways in which they have been addressed in England, France and Germany.

In England, the implementation of genomic medicine is largely driven by Genomics England (GEL). GEL is a British company owned by the UK Department of Health and Social Care and set up in 2013 to run the 100,000 Genomes Project (100K GP). Subsequently, in partnership with GEL, the National Health Service (NHS) established the NHS Genomic Medicine Service (GMS) which provides whole-genome sequencing (WGS) and other genetic tests to NHS patients. New initiatives include the pilot phase of the Newborn Genomes Programme, COVID-19 Study and the Diverse Data Initiative. In France, the Plan France Médecine Génomique 2025 (PFMG) was launched in 2015 with the mission to ensure access to genomic medicine for everyone. It is piloted by AVIESAN, an alliance regrouping the main stakeholders of life and health sciences in France. The PFMG is supported by the government to position France among the leading countries involved in genomics by sequencing 235,000 genomes per annum between 2020 and 2025. Finally, in Germany, GenomDE, an initiative for establishing a nation-wide genome sequencing platform, aims to integrate genomic medicine into routine healthcare. Since 2019, it has begun to create a secure database system linking healthcare and genomic research data and is planning to start a pilot project in January 2023. The German Human Genome-Phenome Archive (GHGA) seeks to provide this nation-wide resource for archiving, accessing, and sharing patient genomes and related omics data, currently mainly for research purposes.

While each national strategy strives to integrate genomic medicine into their public healthcare system to primarily benefit patients, there is also an increasing emphasis on the importance of fostering industry, innovation, and economic growth. This new focus can present challenges to public trust in their national public healthcare systems to serve the good of patients and the public, especially when commercial interests enter the domain of health data governance [8]. We compare how each country is governing data collection and management as a way to (re)negotiate the social contract with the goal of maintaining public trust in data governance. By ‘trust’, we refer to the foundation on which the very legitimacy of democratic governance rests, and as such is crucial for ensuring the success of a wide range of public policies [9]. Consequently, we discuss different models of data governance including data collection, privacy and consent modalities, and data management and access plans.

## Data collection, privacy and consent modalities

### The English Model: from 100,000 to 5 million genomes and beyond

The UK government launched the 100K GP in 2013. When the Project completed its sequencing target in 2018, a further ambition was announced to analyse five million genomes by 2023. Building on evidence, the outcomes of the 100K GP have been transitioning to routine clinical care. Since 2021, the NHS GMS offers patients both the benefit of genomic testing (diagnoses and investigating risk) in clinical care as well as the option to participate in research to develop new treatments. All patients and relevant relatives offered WGS testing are asked if they want to donate their sample (blood, tissue, etc.), genome sequence and health data for research by permitting access to data via a secure national standardised research resource, the National Genomic Research Library (the Library). The Library allows researchers to access pseudonymised genomic and other associated health data (not attributable to specific identifiable data subjects) to carry out analyses within a secure research environment.

Consent materials have been produced in consultation with stakeholders, including clinicians and patient/research participant representatives to enable a broad approach to consent where participants can choose whether to receive a specific set of additional findings [10]. Consent may be recorded via electronic or paper-based means as long as the information is duly provided and communication offered [11]. The information and consent documents have been developed by the GEL ethics team, and with the advice of the Ethics Advisory Committee, the Participant’s Panel, the Science Committee and other stakeholders [12]. Stakeholder engagement is an important part of the Library’s activities, whose aim is to promote understanding and transparency and thus foster trust with patients/participants and the general public.

Participants who consent to have their data accessed via the Library agree to deposit data (and samples) (i) for use in approved research in linked, pseudonymised format; (ii) to be re-contacted and invited to further research; (iii) for the communication of clinically applicable research results via the NHS GMS. In addition, participants give consent for linked access to their health records. The Library also pays attention to the involvement of children, young people and adults who lack or have subsequently lost capacity by seeking the advice of appropriate consultees regarding their participation. Finally, genomic testing is also offered to relevant relatives to refine diagnosis (trio testing), or because they may benefit from the testing. Healthcare professionals have a duty to weigh the interests of genetic relatives in balance with the interest of maintaining the confidentiality of the primary patient [13]. The General Medical Council considers that confidentiality is not absolute, and if a patient refuses to consent to information being disclosed that could benefit others, disclosure might still be justified ‘in the public interest if failure to disclose the information leaves others at risk of death or serious harm’ [14]. The Joint Committee on Genomics in Medicine further argues that disclosure to relatives might be possible in some circumstances without any breach of confidentiality [15].

### The French Model: establishing a governance framework for genomics

Launched in 2015, the PFMG evolved from a request to AVIESAN by the then Prime Minister. From the standpoint of an overall governance structure, the PFMG has established two high-throughput genome sequencing platforms, as well as a centralised system for data analysis, the Data Collector Analyser (DCA). The DCA is the main infrastructure for collecting, analysing, and assisting in the interpretation of genomic data at a national level. Its role is to process, analyse and use the data for either care or research protocols.

The PFMG has also designed four pilot programmes in the realm of cancer and rare diseases, common diseases (diabetes), intellectual disability and genomic diversity within the general French population. In accordance with existing legislation, the DCA will host all data provided and ensure the management of data from major national and international genomic medical research projects.

Finally, the PFMG created the Centre of Reference, Innovation, eXpertise and transfer (CRefIX) [16]. This organism serves as a research think tank hub responsible for integrating future technological innovations into the PFMG. It is also tasked with developing procedures and harmonising protocols and methods. Ensuing projects in innovation will then be implemented through public-private partnerships with the creation of a new associated sector between the two entities. CRefIX will integrate new practices via specific training modules in accordance with necessary regulatory and ethical developments. Finally, CRefIX, in collaboration with the French National Human Genome Research Centre, oversees the sequencing and primary bioinformatics analyses of all projects launched.

From the standpoint of information, privacy, and consent modalities, the PFMG must abide by the most recent revisions of the French Bioethics Laws voted on 2 August 2021 [17]. Indeed, some of the articles of the revised laws have an impact on the information, consent forms and privacy concerns of patients. Firstly, current consent forms must be updated to consider incidental findings and explain their impact on patients and their relatives. Each person will have the choice of whether to be informed of genetic characteristics unrelated to the initial indication for testing. However, if their family members could benefit from prevention measures, this information must be passed on to them.

The situation where a person is unable to express his/her wishes or is deceased has also been modified: the examination may be undertaken for medical purposes in the interest of genetic relatives.

All of these changes voted by the French Parliament still await governmental decrees in order to be implemented, as well as the establishment of ‘Good Practices’ by the Agence de biomédecine (national agency for the oversight of biomedical research and care).

### The German Model: centralising scattered data

Unlike the English and French initiatives, there are no large integrated genome resources in Germany. Genomic and other omics data are scattered across different private and public research institutions and laboratories, and often stored in a decentralised, project-based, and temporary manner. Until recently it has been difficult for researchers to find the data they require, or to safely share the data they have generated. In 2020, the National Research Data Infrastructure (NRDI) was established to make these data systematically available and usable to researchers and scientists, and provide long-term data storage, backup, and accessibility. One of the NRDI’s consortia is the GHGA whose mission is to: collect omics data from patients, make them easier to find and be used by researchers, and provide opportunities for new and novel Big Data techniques that translate research findings into clinical routine. Ultimately, it aims to develop genomic medicine in Germany, which, due to both restrictive policies and public concern regarding the country’s eugenic past, is lagging behind other countries in genetic diagnostic capabilities [18].

Against this background, and to ensure public support and trust, the GHGA has developed a rigorous data governance structure aimed to address legal and ethical requirements. One of the most important conditions for collecting, storing, and sharing genomic data, is valid consent from those whose data is used and accessed within the GHGA. Consent that was obtained in the context of the original study or clinical investigation is considered as insufficient, and extended consent integrating GHGA-specific modules such as key information about the initiative and its data governance policies is required [19]. The GHGA pays great attention to the compatibility of its policies with original consent models as well as the GDPR, e.g., regarding the duration, location of data use, and transparency about the absence of direct benefit to those donating data, and secondary use of these data outside the original research or clinical context.

The GHGA has developed a toolkit containing different consent modules for clinicians, researchers and institutions desirous to submit omics data to the GHGA. The modules can be integrated into existing consent documents to inform patients and research participants about the possibility of sharing their data with the GHGA.

## Data management and sharing

### The English Model: reading not lending data

The NHS Research Ethics Committee approved protocol for the Library is compliant with a range of laws and regulations concerning the use and management of personal data to protect privacy and confidentiality (e.g., General Data Protection Regulation 2018, Data Protection Act 2018). The commitments made to data subjects are iterated in the consent forms that are submitted with a protocol for ethics approval.

GEL and the Library have established a system of allowing data access by approved researchers or private entities. This is mediated via a ‘Data Access Committee’ which, in addition to scientific expertise, has participant representation and an independent chair [20].

All patient data are held in secure facilities in the UK. The structure relies on a cloud service from Amazon Web Services based in the UK. All data from the Library stays within this secured environment where it can be analysed, but not downloaded.

All researchers who wish to access the data, whether from for-profit or non-profit organisations, must apply to the Access Review Committee and either be affiliated with an academic institution which has already signed a participation agreement with GEL or, in the case of commercial entities, sign a Data Access Agreement.

Only summary data can be extracted from the Library for specific and preapproved uses (e.g., presentations/publications) via the Airlock. This system is designed to restrict the output of data information from GEL and thus encourage public trust. Indeed, researchers can look at data and ask questions, but they can only take away the answers to their own research questions expressed in their request for access [21].

In this way, GEL considers the Library - designed under the auspices of the ‘Trusted Research Environment’ concept—‘to be a reading library, not a lending library’. In other words, researchers work only within the research structure and cannot export individual-level data.

### The French Model: building data-sharing collaborations

An important commitment of the PFMG in the sharing of data aims both at the direct interests of patients and those of French research and France’s own economic standing in international competition. Indeed, in the context of data sharing, the PFMG has already participated in numerous and robust sharing programmes. For example, at the international level, a bi-lateral agreement was signed in 2018 between France and Great Britain [22] to develop a joint normative framework ensuring that both partners adopt new technologies appropriately while also advancing in both research and care pathways. The PFMG is also working on the implementation of collaboration agreements with Genome British Columbia and Genome Quebec (Canada).

On a larger scale, efforts are underway by the European Commission to establish a European Health Data Space for the sharing of genomic and clinical data [23]. Following a call for proposals, the consortium led by the French Health Data Hub (HDH) was chosen to establish this pilot programme. Among other objectives, this project is tasked with designing and implementing a comprehensive communication strategy aimed at informing citizens. However, unlike the English and the German model (see below), France has not yet established a robust public engagement initiative with the objective of sharing principles and values so to promote trust, a key pre-requisite for success in this endeavour.

### The German Model: a two-tier model separating research and clinic

As aforementioned, two national structures are currently being set up to advance genomic medicine in Germany: the GHGA for omics and related data primarily generated in research/clinical trials, and GenomDE for quality assurance and data infrastructure of gene panels and WGS done in routine healthcare. GHGA will collect, harmonise, securely manage and provide access to data according to the FAIR principles [24]—making data Findable, Accessible, Interoperable, and Reusable—whilst meeting Germany and Europe’s data protection requirements [25]. As a data archive, GHGA not only receives data from major sequencing research centres but also serves as the German access and entry point for the European Genome-Phenome Archive. Record linkage to the German Medical Informatics Initiative (MII), rendering clinical data accessible for secondary use in research [26] and to genomic data generated within the genomDE infrastructure, are still under debate.

The ELSI (Ethical, Legal, and Social Implications) team is involved in the development of relevant documents and consent modules [19] to ensure that ethical and legal expertise is embedded in GHGA’s strategies from the start. An important aspect of this workstream is the involvement of patient/participant representatives in the conception and governance of GHGA data management plans. The aim is to build and maintain trust among patient groups as well as the public at large, through transparency, accountability, reliable oversight, and exchange of perspectives. With this aim, a qualitative study using deliberative democratic forums with patient representatives as co-researchers has developed information material for data donors and explores what type of concrete roles patients see themselves taking and whether or how these can be operationalized [27]. The second national structure, GenomDe, seeks to enable the integration of genomic medicine into healthcare by uniform, quality-assured and standardised diagnostics provided by specialised centres. GenomDe is legally regulated by a new paragraph introduced into the Social Code Book V §64e as a model-project to regulate reimbursement of the service, and is limited to rare disease and cancer patients over a 5-year test period from 2023. However, to date it is not clear how and whether the two structures—one managing omics data generated in clinical contexts (GenomDE) and other archiving genome sequences generated in research setting (GHGA) will be linked.

## Re-visiting the social contract

Each of the strategies thus far discussed aims to adapt their legal, ethical and governance frameworks to respond to new concerns raised by large-scale health data collection and management in each country. In so doing, each initiative has attempted to (re)define arrangements and expectations that govern their relationship with patients/participants, and more broadly with the public [28]. Indeed, further to the idea that trust serves as a foundation for the social contract and democratic governance (and vice versa), trust as an ethical principle requires that citizens are informed about and understand what they agree to. This means that transparency and accountability (i.e., trustworthiness) becomes a necessary ethical norm in the case of data collection, sharing, and storage [29, 30].

Historical, political and cultural factors have had a decided impact on how each system responds to the challenges raised in the context of large-scale health data. For example, the historical and cultural emphasis placed by the UK on the individual and their exercise of autonomy, has led public authorities to gather input from individual stakeholders before determining public policy via upstream public debates, and exchanges with patients and the public. Similarly, in Germany, specific contours of its history in medical research and treatment of persons during the Second World War bear upon decision making in the realm of genomics [18]. Indeed, public consultations and patient/participant involvement must play an important role in the development of policy. This is not to say that in France such endeavours do not exist, but examples are fewer. French public authorities tend to rely more on experts and high-level specialised public servants, which reflects the top-down governance functioning of the French State. Indeed, France remains a centralised state where the most important decisions and public policy-making emanate from the national executive and legislative branches.

### Common challenges raised by large-scale health data collection and management

Apart from specific challenges faced by all three countries, an important analysis of recent norms relative to genomic data management practices and principles must be accounted for. One example among others is the ‘hybridised’ nature of genomics and blurred boundaries between clinical care and research [1], one which may leave patients and research participants often uncertain about what kind of results they can expect and the meaning of their participation [2]. Indeed, it is often unclear for patients/participants relative to what research realm their data are used for and who will have access to them. Examples where large genomic databases are used to establish genetic correlations with social factors clearly demonstrate that participants can be concerned about the use of the data [31]. This makes it complex for all stakeholders to develop appropriate consent procedures that are both sufficiently specific and broad at the same time. It can also create tensions seeing that established values such as individual choice regarding the activity to be involved in, the data to be shared and the results to be returned, cannot always be fully respected.

Furthermore, the fact that data-driven healthcare such as genomic medicine promises to contribute not only to the best available patient care but also to industry and economic growth, generates the potential for concerns about the motivation of creating large-scale data resources and whether patient benefit remains the primary aim. Also, the promised contribution to the industry implies partnerships with commercial companies, which raises concerns about privacy protection where data leaves the initial environment and/or is accessed by third parties [32]. Indeed, the relationship with for-profit companies and the need to work out what is appropriate raises a plethora of questions about patient and public trust and their expectations of their healthcare systems [8].

### Future challenges for each country raised by large-scale health data collection and management

As the three countries attempt to respond to the aforementioned issues raised by large-scale health data collection and management, they also encounter their own specific challenges. In the case of GEL, the transition from the 100K GP to NHS GMS, the integration of new cohorts (e.g., new-born’s sequences) and the increasing complexity of data export requests raise concerns about the robustness and appropriateness of the Library protocol. Despite the initially adopted broad consent model, questions arise as to whether this model is still sufficient to ensure privacy and valid consent, or whether additional consent is needed for the extended activities. This is particularly important as research [10] has shown that participants in the 100K GP did not always understand the complexity of the project or the specifics of what they had (or not) consented to. The study also reported misunderstandings about the kind of results patients might receive, and how these might affect them or their relatives. The authors of the study argued that rather than covering everything in one specific, although broad, consent discussion, some adaptation to what is most important to the individual making these decisions, is important. Indeed, broad consent will often need to be bolstered by trustworthy systems demonstrating that data is used in a responsible way [33, 34].

In France, the collaboration between the PFMG and the HDH has recently raised reservations expressed by the Commission Nationale de l’Informatique et des Libertés (CNIL), responsible for ensuring the full protection of the security of data made available and of donor anonymity. CNIL’s criticism mainly points to the lack of oversight of the HDH’s security and the fact that it transfers data outside the EU, using the storing services of the US company Microsoft to electronically host the health data. This means that data control does not remain solely in the hands of the HDH responsible for controlling the security conditions of the data they are entrusted with [35].

Indeed, the European Court of Justice ruled that surveillance carried out by American intelligence services on the personal data of European citizens was insufficiently regulated and without any real possibility of appeal [36]. It concluded that transfers of personal data from the EU to the US are contrary to the GDPR and the EU Charter of Fundamental Rights unless additional measures are put in place. Likewise, the French Conseil d’Etat recognised the risk of legal incompatibility as well as weak data privacy protection outcomes [36]. The CNIL further insisted that hosting and related services be reserved only for entities that come under the exclusive jurisdiction of the EU. So far, the French government has established the possibility to file suit under the GDPR for illegal access requests. Many concerned stakeholders are demanding more rigorous upstream data protection safeguards to secure trust among citizens and convince them of the HDH’s commitment to improve quality of care and support for patients [37].

In Germany, one of the main challenges is the linkage of GHGA to the German MII which makes clinical data accessible for secondary use in research, and to genomic data generated within the GenomDE infrastructure. To date, these structures are developed separately with different governance frameworks and it is not clear whether and how the three structures (MII, GenomDE and GHGA) can be intergrated [19]. Furthermore, the effort of GHGA is to centralise data access for omics research in a strongly decentralised, federal country where many policy decisions are regulated at a state (Länder) level rather than at a national level. Currently, legislation and arrangements for data privacy oversight (e.g., whether and what kind of informed consent is required) varies from federal state to state making a competitive digitalisation of health data difficult [38]. A system where dialogue and coordination of national data resources are to be constructed ought to develop rigorous policies to gain public trust and convince citizens of its commitment to benefit patients and the society at large [39].

## Conclusion

The prospects of improving individualised patient healthcare as well as contributing to the scientific and research prosperity of any given country engaged in health data collection, storage and processing are undeniable. However, as our overview has shown, large-scale health data collection and management raise important questions about what is reasonable for citizens to expect of scientists and their health system, both in terms of the use and careful management of their biomedical data. In other words, challenges are posed to the social contract between society and science/medicine, and public trust. Firstly, it is necessary that any governance and data management programme be transparent, accountable, and clearly communicated to patients/participants and citizens. If patients/participants are uncertain about what they can expect in terms of benefits, outcome, and future usage of their data, their consent then appears to be unsatisfactory from a legal and normative standpoint. In addition, when third parties partake as stakeholders or participant data leaves the initial trusted environment, transparent consent protocols come centre stage to assuage the concern that the data are being used more for the benefit of industry than for that of the patients. In this regard, consent processes must tease out and address the different responsibilities that arise in a clinical and research context. Finally, it is important to be aware of how incoherencies between regional, national, and supranational regulations and recommendations raise unresolved issues linked to cross-border data transfer.
